# Value of DWI volumetry for assessment of complete rectal cancer response after CRT

**DOI:** 10.1186/1470-7330-15-S1-P47

**Published:** 2015-10-02

**Authors:** M Nazar, A Vazquez, L Alarcon, M Pascuzzi, M Wirtz, E Eyheremendy

**Affiliations:** 1 Hospital Aleman, Buenos Aires, Argentina

## Learning objectives

To determine the diagnostic performance of DWI volumetry for the assessment of complete response after CRT in patients with locally advanced rectal cancer and to compare with volumetry on standard T2- weight MRI, by means of volumetric signal intensity measurements.

## Content organisation

We retrospectively analyzed 25 patients with locally advanced rectal cancer. Patients underwent pre and post-CRT standard T2 weight MRI and DWI MRI. We placed free-hand regions of interest and each tumour-containing section to determine pre and post-CRT tumour volumes.

Histologic findings were the standard of reference.

## Conclusion

Volumetry of the viable tumour remmants based on signal intensity characteristic on DW images after CRT was shown to be more valuable than volumetry of the tumour based on morphologic T2w images for the differentiation between a pCR and residual tumour in locally advanced rectal cancer.

